# Understanding Multi‐Victimization: Identifying Socioecological Supports Among Adolescents

**DOI:** 10.1111/josh.70179

**Published:** 2026-06-24

**Authors:** Emil K. Smith, Rachel E. Gartner, Paul R. Sterzing, Robert W. S. Coulter

**Affiliations:** ^1^ School of Social Work University of Pittsburgh Pittsburgh Pennsylvania USA; ^2^ Clinical and Translational Science Institute, School of Medicine University of Pittsburgh Pittsburgh Pennsylvania USA; ^3^ School of Applied Social Sciences University College Cork Cork Ireland; ^4^ School of Social Welfare University of California Berkeley California USA; ^5^ Department of Behavioral and Community Health Sciences, School of Public Health University of Pittsburgh Pittsburgh Pennsylvania USA

**Keywords:** adolescent, bullying, gender identity, sexual violence, social support, victimization

## Abstract

**Background:**

Transgender and gender‐diverse adolescents (TGDA) experience multiple victimization types (i.e., multi‐victimization) more often than their cisgender peers. While greater socioecological supports are associated with reduced victimization, their role in protecting TGDA against multi‐victimization is underexplored.

**Methods:**

We conducted a cross‐sectional analysis of health risk behavior survey data from students (*N* = 4207) across 13 high schools in a mid‐sized U.S. city. We compared victimization rates and socioecological support levels (i.e., parental monitoring, perceived social support, food and housing security) and associations between socioecological support and victimization, accounting for between‐school differences. We included two‐way interactions between gender and socioecological supports.

**Results:**

TGDA reported more frequent multi‐victimization and lower socioecological support than cisgender adolescents. Across all groups, greater socioecological support was associated with experiencing fewer victimization types. Food security was more protective for cisgender girls than cisgender boys.

**Implications for School Health Policy, Practice, and Equity:**

Enhancing socioecological support may reduce multi‐victimization for all youth, with TGDA having the greatest need. Schools' advocacy and innovative efforts to bolster socioecological support are critical to TGDA wellbeing.

**Conclusions:**

Modifiable socioecological supports may reduce adolescent multi‐victimization. TGDA have lower support rates and higher multi‐victimization rates, suggesting the need for tailored interventions.

## 1 Introduction to Multi‐Victimization Among Adolescents

Adolescent victimization rarely occurs as a single, isolated event. Rather, young people are often exposed to multiple forms of victimization across relationships and settings, including the home, school, peer group, dating relationships, neighborhood, and online environments. This pattern is commonly described as multi‐victimization or polyvictimization and reflects the accumulation of different forms of victimization within a young person's life [1, 2, 3, 4]. A polyvictimization framework is important because research focused on only one form of violence, such as bullying, sexual victimization, or child maltreatment, can obscure the broader social ecology of adolescent victimization [1, 4].

Youth exposed to one form of victimization are at elevated risk for additional exposures, suggesting that violence clusters within particular social and developmental contexts [1, 4, 5]. This cumulative framing matters because the total burden of victimization is often more strongly associated with trauma symptoms, depression, suicidality, and other adverse mental and behavioral health outcomes than any single form of victimization considered in isolation [6, 7]. In one foundational study, polyvictimization was a better predictor of trauma symptoms than any individual form of victimization, including sexual abuse [1].

### 1.1 Disproportionate Risk of Multi‐Victimization Among TGDA

Transgender and gender‐diverse adolescents (TGDA) face disproportionate rates of victimization relative to their cisgender peers, including elevated rates of bullying, sexual violence, physical assault, and other forms of interpersonal harm [6, 8, 9, 10]. For example, trans male adolescents are more than twice as likely (42.6% vs. 18.5%) to experience 20 or more different forms of victimization within their lifetime compared to their cisgender sexual minority male peers [10]. Similarly, approximately half of transgender male adolescents and nearly two‐thirds of transgender female adolescents experienced ten or more different forms of victimization in the past year [9]. These findings suggest that for many TGDA, victimization is not confined to a single relationship, setting, or perpetrator but instead reflects cumulative exposure across contexts [11].

Consistent with the broader literature, multi‐victimization has significant consequences for TGDA, contributing to negative health outcomes such as depression, suicidality, self‐harm, and post‐traumatic stress [12], while also impairing academic performance and school engagement [13]. Despite knowledge of these impacts, research on multi‐victimization with TGDA remains limited, with few studies examining the prevalence of or protective factors against multi‐victimization in this population. Given the profound effects on mental and physical health, academic outcomes, and overall development, understanding potential protective factors against multi‐victimization experienced by TGDA is critical.

TGDA's disproportionate rate of experiencing multi‐victimization should not be understood as an inherent feature of TGDA identity. Rather, it reflects the ways that anti‐trans stigma is organized across structural, interpersonal, and familial contexts. TGDA must often navigate institutions and environments that stigmatize gender diversity, lack affirming resources, and increase exposure to unsafe conditions. Structural discrimination, hostile school climates, exclusionary policies, and barriers to affirming services can heighten victimization risk while limiting access to the very supports that might interrupt it [3]. At the interpersonal level, family rejection, peer hostility, and chronic stigma‐related stress may further increase the likelihood that victimization accumulates across settings [8, 9].

Prior work also suggests that these risks are embedded within broader social ecological conditions. Among TGDA, family‐level transnegative microaggressions and peer rejection have been associated with greater polyvictimization risk [9]. Material hardship may further intensify this vulnerability. Housing instability and food insecurity can increase adolescents' exposure to unsafe relationships and environments while reducing their ability to avoid or exit harmful contexts [14]. In this sense, the heightened risk of multi‐victimization among TGDA is not simply interpersonal; it is socially produced through interlocking systems that concentrate risk and undermine safety.

A polyvictimization framework is useful because it captures whether TGDA have any reliable context in which they are safe from violence. Consistent with Finkelhor's work on children's exposure to violence, multi‐victimization may signal the absence of a “safe harbor” in a young person's life: a person, relationship, or setting where violence is not ongoing and support is available [4]. For TGDA, who may encounter stigma across multiple contexts simultaneously, the absence of such a “safe harbor” may be especially consequential.

### 1.2 Protective Factors Against Multi‐Victimization

Although the literature on TGDA multi‐victimization remains limited, existing scholarship suggests that socioecological supports may protect against cumulative violence exposure. Protective processes are likely distributed across family, peer, school, and material domains rather than located solely within the individual adolescent. This is consistent with a social ecological understanding of victimization and the growing recognition that prevention efforts must move beyond single‐type victimization models toward broader constellations of risk and protection [2, 7].

Greater parental knowledge, communication, and awareness may reduce adolescents' exposure to contexts associated with higher rates of victimization and increase the likelihood that victimization is noticed and addressed [15, 16, 17, 18]. Perceived social support, including support from peers and non‐parental adults, may also reduce vulnerability by enhancing connection, help‐seeking, and interpersonal safety [19, 20, 21]. These relational supports may be particularly important for TGDA, whose victimization risk is often intensified in the very settings where adolescents might otherwise expect protection (e.g., family settings) [11, 22].

Material supports may also function protectively. Food security and housing security are not merely background socioeconomic conditions; they help shape whether adolescents are shielded from violence or repeatedly exposed to it [23, 24]. Stable housing and reliable access to food may reduce survival‐based vulnerability, limit exposure to unsafe environments, and increase connection to supportive adults and institutions. For example, food and housing insecurity are both associated with higher rates of intimate partner violence among cisgender adults [25], and housing insecurity has been associated with greater risk for intimate partner violence and experiencing a transphobic hate crime among transgender adults [26, 27]. Despite TGDA being at greater risk for food and housing insecurity [28, 29], research has not yet examined how food and housing security are associated with multi‐victimization for TGDA.

Protective factors against TGDA multi‐victimization remain underexamined. Much of the existing literature focuses on single forms of victimization or aggregates sexual and gender minority youth rather than focusing on TGDA specifically. As a result, less is known about whether modifiable socioecological supports, such as parental monitoring, perceived social support, food security, and housing security, are associated with lower levels of multi‐victimization among TGDA.

### 1.3 Current Study

The present study addresses this gap by examining socioecological supports in relation to adolescent multi‐victimization, with particular attention to TGDA. Guided by a social ecological perspective, we conceptualize multi‐victimization as a cumulative pattern of violence exposure shaped by adolescents' relational, institutional, and material environments.

We conducted a cross‐sectional analysis of survey data of high school students from a mid‐sized urban school district in the northeastern United States. Our objectives are to (1) compare the rates of multi‐victimization between TGDA and their cisgender peers, (2) examine the presence of socioecological supports in TGDA's lives and their potential role in mitigating rates of multi‐victimization, and (3) assess whether the association between socioecological supports and multi‐victimization varies among TGDA and their cisgender peers. Given that polyvictimization is one of the strongest indicators of adverse mental and behavioral health outcomes, identifying supports has implications for prevention, intervention, and school health equity [1, 6, 30].

## 2 Methods

### 2.1 Participants

This study surveyed 9th‐12th grade students at 13 high schools in a medium‐sized city in the northeastern United States. The response rate was 71.2%; 4487 surveys were returned; and 280 surveys (6.2%) were excluded due to illegibility, fewer than 20 answered questions, or dishonest response patterns. An additional 1306 participants were excluded from the present study due to skipping key demographic questions or victimization questions, and an additional 979 participants did not answer questions about their support system, making the analytic sample *N* = 2211. Excluded respondents were more likely than included respondents to be trans or gender diverse (*p* < 0.001), of a minoritized race/ethnicity (*p* < 0.001), or attend a predominantly Black school (*p* < 0.001). There was no significant difference between the sexualities of the included sample and the excluded sample (*p* = 0.73).

### 2.2 Procedure

Students who were present on the day of survey administration were eligible to voluntarily participate. This paper‐and‐pencil survey was modeled after the US Centers for Disease Control and Prevention Youth Risk Behavior Survey (YRBS) and was administered during a class period in October 2018. Students' parents/guardians provided passive consent, and students assented to participate in the survey. No compensation was provided for completing the survey. The University of Pittsburgh IRB deemed this cross‐sectional analysis exempt from review. School‐level data for the year in which this survey was conducted (2018–2019) were drawn from the Pittsburgh Public School's website which publicly posts enrollment data.

### 2.3 Instrumentation

#### 2.3.1 Demographics

Gender identity was assessed via the two‐step method [31]. Early in the survey, participants were asked to indicate their sex assigned at birth, as written on their birth certificate, and response options included female or male. Later in the survey, participants were asked to indicate which gender identities best described them by responding yes or no to each of the following identities: girl, boy, trans girl, trans boy, genderqueer, non‐binary, or another identity. Participants could indicate that they identified with multiple gender identities. Youth who indicated that their identity was trans, genderqueer, non‐binary, or another identity and youth whose gender identity did not correspond with the sex they were assigned at birth were categorized as TGDA.

Demographic covariates included sexuality, age, race and ethnicity. Participants could indicate that they identified with multiple racial identities or sexualities. Participants were asked to indicate which sexuality best described them by responding yes or no to the following identities: heterosexual (straight), mostly heterosexual (mostly straight), gay or lesbian, bisexual, queer, asexual, or not sure. Participants who indicated any sexuality other than heterosexual were coded as sexual minorities. Participants were given a list of racial and ethnic identities and asked to indicate all identities that applied. Response options for race included White, Asian, Black or African American, American Indian or Alaska Native, Native Hawaiian or Other Pacific Islander, or Other. A separate question asked students to indicate whether they identified as Hispanic or Latino. For analysis, these responses were combined into the following categories: Non‐Hispanic/Latino White, Hispanic/Latino, Black/African American, Asian/Pacific Islander, American Indian or Alaska Native, Multiracial, and Other. The question assessing age included response options from 12 years old or younger to 18 years old.

#### 2.3.2 Socioecological Supports

Socioecological supports included 4 measures: parental monitoring, perceived social support, housing security, and food security.

Parental monitoring was assessed via the 5‐item child disclosure subscale from Stattin & Kerr's parental monitoring measure [25]. These items (e.g., “You like to tell your parents about what you did and where you went during the evening.”) assessed respondents' openness and secrecy with parents regarding activities during free time, evening and weekends, friendships, and school via 5‐point Likert scales, ranging from “Strongly Agree” to “Strongly Disagree.” This scale had acceptable internal consistency (Cronbach's alpha = 0.76). Individual parental monitoring scores were determined by calculating the mean score of the five items.

Perceived support (including from non‐parental adults) was assessed via the modified Social Support Questionnaire (SSQ6) from Sarason and colleagues [26]. Four items (e.g., “Is there someone you really count on to care about you, regardless of what is happening to you?”) assessed availability and accessibility of social supports and the number of adults other than their parents who they could seek help from via 5‐point Likert scales, ranging from “None of the time” to “All of the time.” This scale had good internal consistency (Cronbach's alpha = 0.82). Individual social support scores were determined by calculating the mean score of the four items.

Structural factors assessed included food and housing security, which were both single‐item measures. To assess for food security, participants were asked “In the past 30 days, how often did you worry that you or your family would have enough food?” Response options included “Never,” “Rarely,” “Sometimes,” “Most of the time,” and “Always.” To assess for housing security, participants were asked to respond “Yes” or “No” to the question, “In the past 12 months, did you have to stay two or more nights in a place that was not your home because you could not stay in their home, you were told to leave, or you did not want to stay in your home?” For both items (food security and housing security), a yes = 1 (housing secure or food secure) and no = 0 (housing insecure or food insecure).

#### 2.3.3 Victimization Experiences

Our dependent variable was a victimization experiences summary score that included sexual victimization, bullying, and physical violence and ranged from 0 (no experiences of victimization) to 3 (experienced all three forms of victimization). An experience of sexual victimization was defined as answering positively to any of the following response items, which were collapsed into a yes/no binary format: (1) “During the past 12 months, how many times did anyone force you to do sexual things that you did not want to do?”, (2) “During the past 12 months, how many times did someone you were dating or going out with force you to do sexual things that you did not want to do?” and (3) “Have you ever been physically forced to have sexual intercourse when you did not want to?” Bullying victimization was assessed through two binary (yes/no) questions: (1) “During the past 12 months, have you ever been electronically bullied?” and (2) “During the past 12 months, have you ever been bullied on school property?” An experience of physical violence was defined as answering positively to any of the following two response items, which were combined into one dichotomous physical victimization variable: (1) “During the past 12 months, how many times has someone threatened or injured you with a weapon such as a gun, knife, or club on school property?” and (2) “During the past 12 months, how many times did someone you were dating or going out with physically hurt you on purpose?”

#### 2.3.4 School‐Level Variables

School‐level variables included enrollment data regarding race/ethnicity and economic disadvantage. Based on the race/ethnic composition of schools, we coded schools as either predominantly white (6 schools) or predominantly Black (7 schools). Economic disadvantage was coded as the proportion of students in each school identified as economically disadvantaged, which is defined by the state of Pennsylvania as students who qualify for state educational tax credits. The proportion of students identified as economically disadvantaged ranged from 5% to 91%.

### 2.4 Data Analysis

We conducted analyses in Stata version 19 [32] and set statistical significance at 0.05. A likelihood‐ratio test indicated that a multi‐level model, accounting for the clustering of students within schools, significantly improved model fit (LR chi^2^ = 17.33, *p* < 0.001).

We conducted cross‐tabulations to describe victimization, housing security, food security, parental monitoring, and perceived social support by gender. To test differences between gender groups, we used multi‐level logistic regression for dichotomous variables (victimization, food/housing security) and multi‐level linear regression for continuous variables (parental monitoring, perceived social support), both accounting for between‐school differences. To determine statistically significant differences in parental support and perceived social support by gender, we conducted multi‐level linear regression models, accounting for between‐school differences.

To examine multivariable associations of socioecological supports and number of victimization types experienced, we conducted multi‐level ordinal logistic regressions in which responses were clustered within schools, controlling for demographic covariates. Parental support scores and perceived support scores were centered around the mean prior to analysis. To examine the moderation effect of gender on the association between socioecological supports and number of victimization types, our final model included two‐way interactions between gender and socioecological supports.

## 3 Results

### 3.1 Demographics and Multi‐Victimization Prevalence

Table 1 shows the demographic characteristics of the 2211 students in the analytic sample. Cisgender girls comprised 54.9% of the sample (*n* = 1631), followed by cisgender boys (38.0%; *n* = 840) and TGDA (7.2%, *n* = 158). The mean sample age was 15.7 years (SD = 1.2). The sample was 48.9% non‐Hispanic/Latino white (*n* = 1081), 25.2% non‐Hispanic/Latino Black/African American (*n* = 556), 11.0% multiracial (*n* = 354), 8.0% Hispanic/Latino (*n* = 177), 4.3% Asian/Pacific Islander (*n* = 94), 0.7% Native American (*n* = 16), and 2.0% other racial/ethnic identities (*n* = 22). The sample was majority heterosexual (67.9%; *n* = 1502) with the remaining 32.3% (*n* = 709) identifying as sexual minority.

**TABLE 1:** Demographics of the analytic sample (*N* = 2211).

	*n* (%)
Gender identity	
TGDA	158 (7.2)
Cisgender Boy	840 (38.0)
Cisgender Girl	1213 (54.9)
Age	
(12–18) Mean (SD)	15.74 (1.2)
Race or ethnicity	
White	1081 (48.9)
Hispanic/Latino	177 (8.0)
Black/African American	556 (25.2)
Asian/Pacific Islander	94 (4.3)
Native American	16 (0.7)
Other	22 (2.0)
Multiracial	243 (11.0)
Sexuality	
Heterosexual	1502 (67.9)
Sexual Minority	709 (32.1)

Abbreviation: TGDA, transgender and gender‐diverse adolescents.

Rates of experiencing multi‐victimization varied significantly across genders (Table 2), with a greater proportion of TGDA experiencing all three victimization types (8.9%) than cisgender girls (3.4%) and cisgender boys (1.1%). TGDA had 4.00 times the odds of experiencing additional victimization types compared to cisgender boys, and TGDA had 2.38 times the odds of experiencing additional victimization types compared to cisgender girls.

**TABLE 2:** Prevalence of number of victimization types by gender identity (*N* = 2211); Pittsburgh, Pennsylvania, 2018.

	No victimization *n* = 1318	One victimization type *n* = 613	Two victimization types *n* = 216	Three victimization types *n* = 64	OR	[95% CI]	*p* ^a^
	*n* (%)	*n* (%)	*n* (%)	*n* (%)			
Gender identity							
Cisgender boy	563 (67.0)	211 (25.1)	57 (6.8)	9 (1.1)	[referent]		
Cisgender girl	695 (57.3)	351 (28.9)	126 (10.4)	41 (3.4)	1.66^b^	[1.38, 1.99]^b^	< 0.001^b^
TGDA	60 (38.0)	51 (32.3)	33 (21.0)	14 (8.9)	4.00^b^ 2.38^c^	[2.84, 5.51]^b^ [1.74, 3.27]^c^	< 0.001^b^ < 0.001^c^

Abbreviation: TGDA, transgender and gender‐diverse adolescents.

^a^ *p* values derived from multi‐level mixed effects ordered logistic regressions adjusting for clustering of students within schools.

^b^ Compared with cisgender boys.

^c^ Compared with cisgender girls.

### 3.2 Multi‐Level Adjusted Models

#### 3.2.1 Prevalence of Socioecological Supports

Compared to their cisgender peers, TGDA experienced the lowest rates of socioecological support across all categories (Table 3), with significantly lower parental monitoring and perceived social support scores than cisgender girls (*p* < 0.001), significantly less food security than cisgender boys (*p* < 0.001) and cisgender girls (*p* < 0.01), and significantly less housing security than cisgender boys (*p* < 0.05).

**TABLE 3:** Prevalence of socioecological supports by gender (*N* = 2211)^a^.

Socioecological support	Cisgender boy (*n* = 840)	Cisgender girl (*n* = 1213)	*b*	[95% CI]	*p* ^b^	TGDA (*n* = 158)	*b*	*p* ^b^
Parental Monitoring (1–5), mean (SD)	3.23 (0.9)	3.51 (0.9)	0.31^c^	[0.24, 0.39]^c^	< 0.001^c^	3.11 (0.9)	−0.04^c^ −0.35^d^	0.575^c^ < 0.001^d^
Perceived Social Support (1–5), mean (SD)	3.51 (1.1)	3.61 (1.0)	0.14^c^	[0.04, 0.24]^c^	0.002^c^	3.23 (1.1)	−0.17^c^ −0.31^d^	0.067^c^ < 0.001^d^
			**OR**	**[95% CI]**	***p*** ^b^		**OR**	***p*** ^b^
Food Security, *n* (%)			0.84^c^	[0.67, 1.04]^c^	0.108^c^		0.48^c^ 0.57^d^	< 0.001^c^ 0.002^d^
No	194 (23.1)	350 (28.4)				72 (45.6)		
Yes	646 (76.9)	863 (71.2)				86 (54.4)		
Housing Security, *n* (%)			0.76^c^	[0.58, 1.00]^c^	0.047^c^		0.57^c^ 0.76^d^	0.016^c^ 0.186^c^
No	98 (11.7)	195 (16.1)				37 (23.4)		
Yes	742 (88.3)	1018 (83.9)				121 (76.6)		

Abbreviation: TGDA, transgender and gender‐diverse adolescents.

^a^ Adjusted for sexual orientation, age, and race/ethnicity and school‐level variables.

^b^ *p* values, odds ratios, unstandardized beta coefficients, and 95% confidence intervals were derived using mixed‐effects logistic regression models for dichotomous variables and mixed effects linear regression models for continuous variables, both of which accounted for differences between schools.

^c^ Compared with cisgender boys.

^d^ Compared with cisgender girls.

#### 3.2.2 Multivariable Model for Main Effects Only

Overall, greater socioecological supports were significantly associated with lower adjusted odds of experiencing multi‐victimization. Participants who reported past month food security had 46% lower adjusted odds (95% CI: 0.45–0.67) of experiencing additional victimization types compared to students who reported food insecurity in the past month. Participants who reported housing security in the past year had 50% lower adjusted odds (95% CI: 0.40–0.64) of experiencing additional victimization types compared to participants who reported housing insecurity in the past year. For each unit increase in parental monitoring, the adjusted odds of experiencing an additional type of victimization decreased by 23% (95% CI: 0.70–0.86). Similarly, for each unit increase in perceived support, the adjusted odds of experiencing an additional type of victimization decreased by 13% (95% CI: 0.80–0.96).

#### 3.2.3 Multivariable Model for Main Effects Including Interaction Terms

Table 4 shows model results when adjusting for socioecological supports, school‐level variables, and including interaction terms. Among respondents who reported average parental monitoring and perceived social support scores and food and housing security, TGDA reported 4.5 times higher odds of experiencing additional types of victimization than cisgender boys, and cisgender girls reported 3.0 times the odds of experiencing additional types of victimization than cisgender boys. For cisgender boys, an increase in parental monitoring score was associated with a 20% decrease in odds of experiencing additional types of victimization, and the presence of food security was associated with 43% reduced odds of experiencing additional types of victimization compared to cisgender boys who were housing insecure.

**TABLE 4:** Multivariable multi‐level regression model examining association of socioecological supports and victimization (*N* = 2211)^a^.

Demographics	OR	[95% CI]	*p*
Gender identity^b^			
Cisgender Boys	1.00	[referent]	
Cisgender Girls	3.03	[1.75, 5.24]	< 0.001
TGDA	4.50	[2.01, 10.08]	< 0.001
Socioecological supports			
Parental Monitoring	0.80	[0.66, 0.95]	0.014
Perceived Social Support	0.87	[0.75, 1.01]	0.062
Food Security	0.78	[0.56, 1.11]	0.165
Housing Security	0.57	[0.37, 0.88]	0.012
Interactions by gender and socioecological supports^b^			
Parental monitoring × gender			
Cisgender Girls	0.95	[0.76, 1.20]	0.674
TGDA	1.10	[0.71, 1.70]	0.671
Perceived support × gender			
Cisgender Girls	1.04	[0.86, 1.26]	0.671
TGDA	0.77	[0.54, 1.09]	0.140
Food security × gender			
Cisgender Girls	0.56	[0.36, 0.85]	0.007
TGDA	0.75	[0.37, 1.53]	0.430
Housing security × gender			
Cisgender Girls	0.84	[0.50, 1.43]	0.525
TGDA	0.76	[0.32, 1.78]	0.526

Abbreviations: OR, odds ratio; TGDA, transgender and gender‐diverse adolescents.

^*a*^ Adjusted for demographic co‐variates (age, sexuality, race/ethnicity) and school‐level variables. We used a two‐tier multi‐level ordinal logistic regression model to adjust for clustering of students within schools.

^b^ Cisgender boys are reference group.

Our analysis included four interaction terms: (1) parental monitoring × gender, (2) perceived support × gender, (3) food insecurity × gender, and (4) housing security × gender. Gender significantly moderated the association between food security and multi‐victimization. Specifically, food security was more protective against multi‐victimization for cisgender girls in comparison to cisgender boys (*p* < 0.05). All the remaining interaction terms were non‐significant.

## 4 Discussion

The current study is consistent with other studies that speak to TGDA's heightened multi‐victimization risk [6], with TGDA participants experiencing 2–3 times higher adjusted odds of multi‐victimization than their cisgender peers. Our study sought to extend prior literature beyond the “high‐risk” narrative that situates TGDA's risk in the individual, to an examination of modifiable environmental factors that may be associated with reduced multi‐victimization among TGDA. Consistent with this goal, we found that TGDA and cisgender peers with higher levels of parental monitoring and perceived social support were less likely to be multi‐victimized. Similarly, we found that TGDA who had more food and housing security were less likely to experience multi‐victimization, with a stronger relationship between food security and reduced multi‐victimization among cisgender girls. Notably, TGDA in our study reported lower parental monitoring, social and non‐parental adult support, food security, and housing security compared to their cisgender peers. These findings add to prior literature by examining multi‐victimization as an outcome rather than studying each form of victimization in silos. In addition, the current study examines multi‐victimization and social support for TGDA compared to cisgender peers rather than looking to undifferentiated (presumed cisgender) samples or sexual and gender minority samples.

Notably, gender did not moderate associations between socioecological support variables and experiencing multi‐victimization, with the exception of food insecurity. This finding suggests that socioecological supports function similarly for cisgender and transgender adolescents, highlighting that TGDA's low levels of socioecological support compared to their cisgender peers are the primary issue, rather than socioecological supports having a greater or lesser protective function for TGDA against experiencing multi‐victimization when compared to their cisgender peers.

### 4.1 Implications for School Health Policy, Practice, and Equity

Our findings suggest that building interventions and policies aimed at strengthening parental monitoring, social support, food security, and housing security may be effective to reducing multi‐victimization for all high school students. Further, our finding that TGDA report lower levels of socioecological supports and higher rates of multi‐victimization suggest that they have the greatest need for programs and policies that positively impact these socioecological supports. Nevertheless, U.S. legislation and executive actions at the federal and state level currently inhibit and/or actively prohibit policy or school‐based supportive interventions. For example, executive order (EO) 14,190 bans transgender students from using gendered facilities or participating in sports that align with their gender, bans teachers and school staff from using TGDA students' names and pronouns, requires that staff inform students' parents of TGDA students' gender identities even if this could increase TGDA's risk of harm, and hampers efforts to improve school climate by banning schools from teaching gender identity‐related topics [33]. Noncompliance risks losing federal funding for schools and legal action against individual personnel (i.e., teachers and staff) [33].

For adolescents in need of socioeconomic supports, schools are a frequent site of intervention. For example, nutritional support (e.g., free or low‐cost meals), workforce development, and career preparation are provided in schools. Schools are already difficult environments for TGDA, with TGDA more likely than their cisgender peers to skip school because they feel unsafe [34]. With schools becoming increasingly unsafe due to anti‐trans bathroom policies, which are known to be associated with higher rates of school‐based TGDA‐targeted violence [35], we may see even greater disparities in rates of truancy among TGDA. The increasing hostility toward TGDA in schools limits the impact of any school‐based interventions to promote TGDA's food security, future job prospects, and housing security.

Parental, social, and nonparental adult supports for TGDA are also being eroded by federal and state anti‐trans policies. In addition to EO 14,190 limiting school‐based non‐parental adult support for TGDA, state‐level policies and directives, such as the Texas Department of Family and Protective Services policy to investigate parents who support TGDA's medical transition, contribute to a fearful and pathologizing political climate that discourages family support of TGDA. Although this policy was struck down by the Texas State Court of Appeals in 2024, the Texas Department of Family and Protective Services has petitioned the Texas Supreme Court to review the decision [36]. Even in states without such extreme anti‐trans legislation, the political climate has an impact on parental support for TGDA, as the presence of state‐level non‐discrimination policies is associated with more supportive family environments [37].

With the structural discrimination faced by TGDA in the U.S., resistance and advocacy are gravely needed to protect TGDA wellbeing. In addition, innovative strategies are needed to bolster socioecological supports for TGDA to reduce multi‐victimization inequities. For example, schools may consider developing partnerships with institutions that are more insulated from federal or state oversight and funding, such as trans‐inclusive religious institutions and privately funded youth serving organizations, as these remain promising sites for bolstering social and emotional support for TGDA, their families, and non‐parental allies. Additionally, TGDA desire explicitly inclusive TGD‐led programs to address housing and food insecurity [38]. Given that the TGD community has a long history of building social networks and support systems to meet each other's needs in the absence of institutional resources, it may be possible to facilitate or support efforts that are already TGDA‐led. Peer support [39], chosen families [40], and mutual aid [41] can be instrumental in facilitating TGDA's access to social support, food, and housing. Despite this, TGDA health research has yet to fully integrate these types of resources into our understanding of strategies to address TGDA's material and social support deficits.

### 4.2 Limitations

This study is not without limitations. These data were collected prior to the COVID‐19 pandemic, so may not accurately reflect how access to socioecological supports may have changed during or after COVID‐19. Cross‐sectional data cannot indicate causality between socioecological supports and multi‐victimization. Although the literature suggests that socioecological supports are protective against multi‐victimization, it has also been suggested that experiencing victimization can lead to reduced socioecological supports among TGDA [12]. Our study is also prone to response and nonresponse biases, although research supports the utility of anonymous, self‐reported, school‐based surveys to measure health risks [42]. Of note, we found that predominantly Black/African American schools had significantly lower response rates on items related to socioeconomic supports than other schools. The varied rates of nonresponse may be attributed to variations in how schools distributed the survey, or how much time was allotted for survey completion, as socioeconomic support items were located at the end of the survey. Despite the survey being confidential, participants may not have wanted to disclose sensitive information to avoid potential consequences, such as child welfare involvement, which disproportionately impacts Black communities [43].

There are also several limitations due to the measures used in this study. This measure of multi‐victimization included only three victimization types. Polyvictimization research often uses the Juvenile Victimization Questionnaire to measure more than 30 victimization types [4]. As a result, the current study may be underestimating the rate of multi‐victimization. The housing security measure focused on participants who were unable to stay at home, which may not capture experiences of adolescents who are chronically unhoused or whose families are unhoused.

There are also additional dimensions of gender, beyond that which was collected in our study (i.e., gender identity) that may be related to multi‐victimization. For example, higher‐levels of gender‐nonconformity are associated with higher rates of school violence for transgender and cisgender adolescents [44]. Further, although we were adequately powered to examine differences between TGDA and cisgender adolescents, we were underpowered to examine differences across subgroups of TGDA (e.g., transmasculine, transfeminine, nonbinary). Similarly, we collapsed sexual identities into heterosexual and sexual minority to achieve adequate cell sizes, thus this study could not examine multi‐victimization differences among diverse sexual identities and levels of social support.

Future longitudinal research, with gender, sexual, and racial and ethnically diverse samples is needed to establish causality and understand identity‐based impacts on the relationship between socioecological supports and experiencing multi‐victimization. In addition, further methodological considerations are needed to identify the best ways to measure multi‐victimization prevalence among youth. It may be worthwhile to consider alternative avenues for collecting more comprehensive data regarding victimization types in ways that ensure confidentiality and do not negatively impact youth respondent wellbeing.

## 5 Conclusions

This study addresses important gaps in literature by shifting the focus of the narrative about TGDA youth violence victimization from individual factors to systems of support. Notably, the same socioecological factors that support cisgender adolescents are supportive of their TGDA peers. Challengingly, these supports are significantly less present in TGDA's lives. Current U.S. policies, orders, and actions are striving to further isolate TGDA from interpersonal and structural resources. As researchers, scholars, and practitioners, we must recognize that bolstering TGDA's access to these important socioecological supports will involve addressing multiple systemic barriers intimately tied to their experience of interpersonal and structural discrimination.

## Funding

This work was supported by the National Center for Advancing Translational Sciences, TL1TR001858. National Institute on Alcohol Abuse and Alcoholism, K01AA027564.

## Ethics Statement

The University of Pittsburgh Review Board deemed this study exempt from review.

## Conflicts of Interest

The authors declare no conflicts of interest.

## Data Availability

Data sharing is not applicable to this article as no new data were created or analyzed in this study. Original data are not public and can be requested from the Allegheny County Health Department.
